# Association Between Onsite Outpatient Pharmacy Availability, Medication Fill Rates, and Readmissions in Mental Health Hospitals

**DOI:** 10.7759/cureus.109139

**Published:** 2026-05-18

**Authors:** Shabnam Sood, Beth Darling, Nikhita Loomba, Dhruv Khosla, Gilbert Ramos, Bikash Bhattarai

**Affiliations:** 1 Psychiatry, Valleywise Health, Phoenix, USA; 2 Psychiatry, Creighton University School of Medicine, Phoenix, USA; 3 Psychiatry, University College of Medical Sciences, New Delhi, IND; 4 Biostatistics, Valleywise Health, Phoenix, USA

**Keywords:** medication adherence, onsite outpatient pharmacy, prescription fill rates, psychiatric inpatients, re-hospitalization

## Abstract

Background: Medication non-adherence is common among patients with serious psychiatric disorders and is associated with relapses, adverse outcomes, and rehospitalization. Discharging patients with medications in hand may improve adherence and reduce readmission rates, yet few studies have examined the role of onsite discharge pharmacies in inpatient psychiatric settings. Objective: This study aims to compare discharge medication fill rates and 30-day readmission rates between two inpatient psychiatric hospitals in the same health system, one with an onsite outpatient pharmacy (MV) and one without (DV). Materials and methods: This retrospective chart review included 565 adult patients discharged over a three-month period. Data collected included demographics, psychiatric diagnoses, discharge medications, prescription types (MV, e-prescription, and printed), and 30-day readmission rates. Filled discharge medications were verified via MV, e-prescriptions, payor pharmacy claims, or contact with external pharmacies. Statistical analyses used chi-square tests; p < 0.05 was considered significant. Results: Patients discharged from MV had higher overall prescription-filling rates (265, 88.6%) than those discharged from DV (148, 54.8%) (p < 0.001). Full prescription fill rates were 212 (82.2%) for scripts sent to MV, 64 (77.1%) for e-scripts, and 81 (35.5%) for printed scripts. Thirty-day readmission rates were lower at MV (22, 7.4%) versus DV (32, 11.6%), though this difference did not reach statistical significance (p = 0.075). The verification revealed that the insurance claims data was only 46% accurate, highlighting the importance of direct verification. Conclusions: Patients discharged from MV with medication in hand had significantly higher medication fill rates and significantly lower readmission rates than those discharged with e-prescriptions or printed prescriptions. This finding contributes to a growing body of research indicating that discharging patients with medication in hand is an effective strategy for increasing medication adherence and reducing rehospitalization.

## Introduction

In 2021, mental illness affected about 1.1 billion people globally and was previously estimated to contribute to about 14% of the global burden of disease [1]. The World Health Organization defines adherence to long-term therapy as “the extent to which a person’s behavior, taking medication, following a diet, and/or executing lifestyle changes, corresponds with agreed recommendations from a health care provider” [2]. A 2020 meta-analysis found that 49% of patients with major psychiatric illnesses were non-adherent to medications [3]. Medication non-adherence is associated with exacerbation of illness, poor response to treatment, rehospitalization, reduced quality of life, unfavorable psychosocial outcomes, and increased medical comorbidities [3-6]. Several factors contribute to poor adherence within the healthcare system, including incomplete discharge planning, inadequate patient support, limited medication coverage, and weak therapeutic relationships. Financial factors also play a significant role, including the unaffordability of medications, high healthcare costs, lack of health insurance, and patients' financial constraints [3,7].

Non-adherence is often a hidden issue, partially because physicians tend to overestimate patient adherence. In a study by Stephenson et al., 153 U.S. physicians were asked to assess the adherence of their patients with bipolar disorder and/or schizophrenia [8]. The study revealed that 72% of the patients whose prescription refill records indicated significant non-adherence were nonetheless rated as adherent by their physicians. This finding suggests that although non-adherence is often acknowledged as a broader issue, it remains undetected during individual patient interactions. This highlights the need for alternative methods to estimate true medication adherence to better identify patients at risk of non-adherence-related consequences. Rosen et al. found that those with low or intermediate medication adherence were 2.54 times more likely to be readmitted to a hospital within 30 days of discharge than those with high adherence [9]. Additionally, El Morabet et al. found that 21% of readmissions were medication-related, with 69% of drug-related readmissions being preventable [10].

Patients who have their medications filled prior to discharge from an inpatient facility tend to have lower rehospitalization rates [11,12]. Filling medications prior to discharge reduces challenges with inadequate transportation to a pharmacy and insurance coverage. Medications prescribed at hospital discharge can result in patient harm if there are barriers to accessing the medications or if instructions are misunderstood [13,14]. Ensuring prescriptions are filled prior to discharge can help reduce these risks. Multiple studies have demonstrated that this practice is associated with improved caregiver experiences and a better understanding of discharge medication instructions [15,16]. While having medication in hand at discharge does not automatically translate to improved medication adherence, it is recognized as one strategy to reduce relapses and prevent exacerbation of symptoms. It has also been speculated that ensuring medications are filled at the hospital prior to discharge, along with offering counseling to the patient on proper usage, may help mitigate the risk of preventable adverse drug events resulting from incorrect medication use [17].

Several interventions have been suggested to improve adherence in patients with psychiatric disorders. These include simplifying the medication regimen, effectively managing and treating side effects, reducing the number of doses, and using long-acting injectable drugs [18]. A recent systematic review analyzed interventions to improve medication adherence and found that dose simplification, patient education, electronic reminders, and incentives were the most effective [18]. Psychosocial interventions have also been used, such as motivational interviewing, behavioral training, and family interventions [19]. However, none of the studies reviewed evaluated access to medication as an intervention.

In this county, within the Southwestern United States, the serious mental illness (SMI) system is separated into regional behavioral health authorities (RBHAs) for financial and managed care purposes. To meet the criteria for SMI benefits in this county, all patients must have at least one qualifying diagnosis that results in functional impairment. This entitlement program is managed by a single insurance company that provides coverage for all patients designated as SMI. Collaboration with this payor allowed for the evaluation of claims based on records to aid in determining medication fill rates.

The study was conducted within a large, urban, public safety-net health system that serves a mostly involuntary mental health population with SMI. There are two hospitals in this system. The inpatient behavioral health hospital has an onsite outpatient pharmacy (MV). Patients discharged from MV have case managers, social workers, or family members pick up their medications from the pharmacy prior to or at discharge. The other inpatient behavioral health hospital does not have an onsite outpatient pharmacy (DV), and patients are either discharged with a paper prescription in hand or have their prescription e-prescribed, faxed, or called in to an outside pharmacy.

The primary objective of the study is to compare discharge medication fill rates between the two inpatient psychiatric locations within the same hospital system (DV and MV) to determine whether medication fill rates differ at MV. Second, we examined whether medication fill rates correlated with rehospitalization rates within 30 days of hospital discharge. Readmission was defined as an admission to this hospital system or another local psychiatric hospital in the county for psychiatric reasons only and not for medical reasons.

## Materials and methods

This study was a retrospective chart review of patients discharged from two inpatient behavioral health hospitals within the same health system (MV and DV) over a three-month period, from December 1st, 2023, to February 29th, 2024. The data collected included medical record number, age, sex, psychiatric diagnosis, admission date, discharge date, insurance identification number, hospital location, discharging provider's national provider identifier number, discharge medication list, prescription type (MV, e-prescribed, or printed scripts), and pharmacy claims data for prescriptions filled. Subject data was collected from the electronic medical record system (Epic). Pharmacy claims data were obtained from the managed care organization (the primary payer for the patients included in this study) and from outpatient pharmacies (listed on the discharge summary) where medications were called in or electronically transmitted (e-prescribed). Valleywise Health Institutional Review Board issued approval 2024-026.

Inclusion and exclusion criteria

The study included patients discharged from the behavioral health hospital's MV and DV from December 1st, 2023, to February 29th, 2024, with SMI insurance as their primary payer. Data were collected for all psychotropic medications (both scheduled and as-needed ones, if prescribed), and data for non-psychotropic medications were not included.

The study excluded patients under the age of 18, patients whose primary payor was not the local SMI insurance, patients who were stepped down to a subacute or other type of inpatient facility where an inpatient pharmacy prescribed medications, patients who were prescribed only a long-acting injectable medication, and patients who were not prescribed any medication upon discharge. Readmission to this hospital and to local hospitals in the county was excluded if they were for medical reasons.

Subject data were obtained from an Epic report, and a request was then sent to the payor for claims-based data on prescriptions filled and hospital readmissions during the study period. To verify the accuracy of the managed care organization data, we compared it with the MV data retrieved directly from Epic. We noted significant inaccuracy in the managed care organization data, which reported both medication fills that did not happen and failed to report fills that were confirmed. Due to these discrepancies, we moved to verify all prescription fills for which the pharmacy was known (MV fills and e-prescribed medications). Unfortunately, verification was not possible for printed prescriptions, as they could be filled at any pharmacy. This method allowed us to determine the accuracy rate of the managed care organization data. We tracked each psychiatric medication prescribed at discharge and categorized patients as having none filled, partially filled (some medications filled), or filled.

Statistical analysis

Data was analyzed using SAS version 9.4 (SAS Inc., Cary, NC, USA). Categorical variables were summarized as counts and percentages. Statistical differences were tested by the chi-square test or Fisher’s exact test for bivariate association. Relative risks and adjusted relative risks, based on log-binomial models, are also presented for readmission and prescription non-filling. A p-value of <0.05 was considered statistically significant.

## Results

Of the 588 initial patients, 23 were excluded from the study because they were prescribed only long-acting injectable medication.

A total of 565 patients were analyzed in the study: 295 (52.2%) at MV and 270 (47.8%) at DV. The most common diagnoses were bipolar disorder (302, 53.5%), schizophrenia (120, 21.2%), schizoaffective disorder (42, 7.4%), major depressive disorder (38, 6.7%), and others (63, 11.2%). There were more males (312, 55.2%) than females (253, 44.8%), and ages ranged from 18 to 79 years with a mean age of 35.5 years.

Among all patients included in the study, 258 (45.7%) had medications filled by MV, 228 (40.4%) received printed scripts, and 83 (14.7%) received e-prescriptions. Four patients at MV had both scripts sent to MV and e-prescriptions, resulting in a higher number of prescriptions (N = 299). MV had 258 (86.3%) scripts sent to MV, 21 (7.0%) e-prescribed, and 20 (6.7%) given as printed scripts. At DV, 208 (77.0%) of prescriptions were printed scripts, and 62 (23.0%) were e-prescribed.

Prescriptions were defined as filled (all psychiatric medication fully dispensed), partially filled (one or more medications not dispensed), or not filled (no medication was dispensed). Medications were filled in 212 (82.2%) of scripts sent to MV, 64 (77.1%) of e-scripts, and 81 (35.5%) of printed scripts across both locations (Table 1).

**Table 1:** Fill rates of different prescription types * 125 printed scripts were unverified (14 for MV and 111 for DV).

Prescription type	Filled n (%)	Partially filled n (%)	Not filled n (%)	Total
In-house	212 (82.2)	30 (11.6)	16 (6.2)	258
E-prescribed	64 (77.1)	7 (8.4)	12 (14.5)	83
Printed*	81 (35.5)	19 (8.3)	3 (1.3)	103
Total (all prescription types)	357	56	31	444

MV had a higher percentage of scripts that were filled or partially filled (265, 88.6%) than DV (148, 54.8%). The difference in fill rates between the two locations was statistically significant (p < 0.001) (Table 2).

**Table 2:** Fill rates of prescriptions by location * N is 299 at MV since four patients had two types of scripts. Chi-square test p < 0.001. MV: with onsite outpatient pharmacy, DV: without onsite outpatient pharmacy

Location	Filled or partially filled, n (%)	Not filled, n (%)	Total
*MV	265 (88.6)	34 (11.4)	299
DV	148 (54.8)	122 (45.2)	270
Total for both locations	413	156	569

We compared the data obtained from the managed care organization on whether prescriptions were filled, partially filled, or not filled, with the data we had verified for each patient. It was noted that payor claims data were only 46% accurate across all prescription types. Of the 228 printed scripts, 125 were unverified (14 for MV and 111 for DV). If 46% of these unverified prescriptions were filled, MV still had a significantly higher frequency of prescriptions filled or partially filled (271, 90.6%) than DV (199, 73.7%). This difference in fill rate between the two locations remained statistically significant after adjustment for unverified prescriptions (p < 0.001) (Table 3).

**Table 3:** Fill rates of prescriptions after adjusting for unverified prescriptions * N is 299 at MV since four patients had two types of scripts. Chi-square test p < 0.001 MV: with onsite outpatient pharmacy, DV: without onsite outpatient pharmacy

	Filled or partially filled N (%)	Not filled N (%)
*MV	271 (90.6)	28 (9.4)
DV	199 (73.7)	71 (26.3)

Relative risks did not change much after adjustment for age, length of stay, and number of antipsychotics prescribed, indicating that these variables do not substantially confound the risk of non-filling (Table 4).

**Table 4:** RR of prescription non-filling by locations * The total is less than n because of missing and unverified prescription information. ** RR adjusted for age, length of stay, and number of antipsychotics prescribed. RR: relative risk, CI: confidence interval, MV: with onsite outpatient pharmacy, DV: without onsite outpatient pharmacy

Location	Not filled, n (%)	Filled or partially filled, n (%)	Total, n*	Unadjusted RR of prescription not filled (95% CI)	Adjusted RR of not filled (95% CI)**
DV	11 (6.92)	148 (93.08)	159	1.02 (0.50-2.10)	1.08 (0.53-2.20)
MV	19 (6.76)	262 (93.24)	281	-	-
Total	30 (6.82)	410 (93.18)	440	-	-

Patients discharged from MV had a lower readmission rate (22, 7.4%) than patients discharged from DV (32, 11.6%) within the study period. This difference was not statistically significant (p = 0.075). The rate of readmission was slightly lower in patients who had filled or partially filled their prescriptions (37, 9.1%) than in those whose prescriptions were not filled or for whom fill status could not be verified (4, 13.3%). This difference did not reach statistical significance (p = 0.45). Relative risks did not change much after adjustment for age, length of stay, and number of antipsychotics prescribed, indicating that these variables do not substantially confound the risk of non-filling (Table 5).

**Table 5:** RR of readmission by location * RR adjusted for age, length of stay, and number of antipsychotics prescribed. RR: relative risk, CI: confidence interval, MV: with onsite outpatient pharmacy, DV: without onsite outpatient pharmacy

Location	Readmitted, n (%)	Not readmitted, n (%)	Total, n	Unadjusted RR of readmission (95% CI)	Adjusted RR of readmission (95% CI)*
DV	32 (11.99)	235 (88.01)	267	1.59 (0.95-2.67)	1.54 (0.92-2.57)
MV	22 (7.53)	270 (92.47)	292	-	-
Total	54 (9.66)	505 (90.34)	559	-	-

## Discussion

Psychiatric hospitalization is an expensive but necessary means to treat patients with SMI who are in a decompensated psychiatric state. At this hospital, most behavioral health patients are admitted involuntarily and leave the hospital with an outpatient court order for treatment. Despite this civil commitment for treatment, patients continue to struggle with medication adherence upon discharge for many reasons, including limited medication access. This non-adherence leads to exacerbation of symptoms, often to the point of dangerousness to self or others, or intractable psychosis requiring hospital readmission.

Many studies have been conducted to evaluate the efficacy of various interventions to improve adherence. They have consistently shown that dose simplification, patient education, electronic reminders, and reduced patient cost sharing or incentives are most effective [18]. No studies were found that examined access to medication on hand upon discharge as a barrier to continuing treatment. Our study demonstrates that having an MV at discharge significantly improves the proportion of patients with medication in hand on discharge, as evidenced by MV patients filling 88.6% of scripts compared to 54.8% at DV (p < 0.001). While 30-day readmission rates were lower at MV (7.4%) than at DV (11.6%), this difference did not reach statistical significance. This finding of a possible correlation between patients not having medications in hand and hospital readmission should be interpreted cautiously, and the lack of statistical significance may be due to the short study period and relatively small sample size. These results align with prior studies suggesting that discharging patients with medications in hand can improve adherence and reduce emergency visits or readmissions. Rosen et al. showed that patients with low and intermediate adherence combined had a readmission rate of 20.0%, compared with 9.3% for those with high adherence. However, they did not compare the method of obtaining medications after discharge [9]. Similarly, Hatoun et al. found that patients discharged with medications in hand had significantly lower odds of returning to the emergency department within 30 days (odds ratio, 0.22) compared with those discharged with usual care [11]. Unlike previous research, our study specifically examines access to medications as a discharge intervention, providing practical evidence that MV is an effective, low-cost strategy to enhance continuity of care.

An additional noteworthy finding was the limited accuracy of insurance claims data. After verification with each pharmacy, it was determined that 46% of the claims data were accurate. At the outset of this project, we expected to use claims-based data as our primary source of verification. Still, due to its unreliability, we had to adjust to verifying each prescription with known pharmacies for each chart reviewed. This finding raises further questions about the accuracy of claims-based data from insurers and how these data points are used to make both global and individual decisions regarding healthcare authorizations.

Limitations include the short three-month period, inclusion of only patients with a specific insurance provider, and the inability to verify all printed prescriptions. These factors may limit generalizability, and the overall adherence and readmission rates may be higher than in other populations.

Despite these limitations, the study offers actionable guidance for inpatient psychiatric services: ensuring medications are available at discharge through MV can substantially improve adherence and may reduce preventable readmissions. Hospitals and clinicians can consider integrating similar strategies, including discharge counseling, home delivery, or enhanced pharmacy coordination, to strengthen continuity of care and improve patient outcomes. Furthermore, the use of checklists and other methods to enhance reliability should be explored to maximize medication fill rates [12].

## Conclusions

Our study demonstrates that an MV significantly increases fill rates and trends toward reduced 30-day readmissions in a real-world inpatient psychiatric population. This finding contributes to a growing body of research supporting the use of in-house hospital discharge pharmacies that provide medications in hand at discharge, potentially reducing the risk of non-adherence following discharge and the consequent risk of relapse in symptoms. This approach can translate to improved patient outcomes, including decreased rates of untreated mental illness and reduced burden on the healthcare system.
